# Simultaneous Measurements of Temperature and Viscosity for Viscous Fluids Using an Ultrasonic Waveguide

**DOI:** 10.3390/s21165543

**Published:** 2021-08-18

**Authors:** Jinrui Huang, Frederic Cegla, Andy Wickenden, Mike Coomber

**Affiliations:** 1 NDE Group, Department of Mechanical Engineering, Imperial College London, London SW7 2AZ, UK; f.cegla@imperial.ac.uk; 2 Rivertrace Ltd., Unit P, Kingsfield Business Centre, Philanthropic Road, Redhill RH1 4DP, UK; awickenden@rivertrace.com (A.W.); mcoomber@rivertrace.com (M.C.)

**Keywords:** ultrasonic waveguide, viscous fluids, temperature, viscosity

## Abstract

The characterisation and monitoring of viscous fluids have many important applications. This paper reports a refined ‘dipstick’ method for ultrasonic measurement of the properties of viscous fluids. The presented method is based on the comparison of measurements of the ultrasonic properties of a waveguide that is immersed in a viscous liquid with the properties when it is immersed in a reference liquid. We can simultaneously determine the temperature and viscosity of a fluid based on the changes in the velocity and attenuation of the elastic shear waves in the waveguide. Attenuation is mainly dependent on the viscosity of the fluid that the waveguide is immersed in and the speed of the wave mainly depends on the surrounding fluid temperature. However, there is a small interdependency since the mass of the entrained viscous liquid adds to the inertia of the system and slows down the wave. The presented measurements have unprecedented precision so that the change due to the added viscous fluid mass becomes important and we propose a method to model such a ‘viscous effect’ on the wave propagation velocity. Furthermore, an algorithm to correct the velocity measurements is presented. With the proposed correction algorithm, the experimental results for kinematic viscosity and temperature show excellent agreement with measurements from a highly precise in-lab viscometer and a commercial resistance temperature detector (RTD) respectively. The measurement repeatability of the presented method is better than 2.0% in viscosity and 0.5% in temperature in the range from 8 to 300 cSt viscosity and 40 to 90 °C temperature.

## 1. Introduction

There are more than 50,000 ships world-wide, which are responsible for transporting over 90% of the globally traded goods, and most of them use Heavy Fuel Oil (HFO) as their main propulsion fuel [1]. The fuel delivery system on these ships requires the temperature and viscosity of HFO to be monitored and regulated for safe and efficient engine management. Conventional methods to determine such viscous fluids include falling ball viscometers and tuning fork resonance-based one, or rotation and torque-based measurements. While many of these methods are cheap and easy to implement, very few of them lend themselves to fast and online monitoring applications.

Ultrasonic waveguides are an attractive alternative due to their potential to provide robust measurements at a relatively low cost. They are suitable for online continuous monitoring and can also be designed to work in harsh environments, such as extremely high temperature and pressure, where conventional techniques are not reliable. For example, Balasubramaniam et al. [2] used a waveguide to measure the viscosity of molten material at extreme temperatures. An experimental setup using ultrasonic waveguides to measure viscosity in high pressure was reported by Kiełczynski [3]. Recently, Liao et al. [4] used dry-coupled waveguide transducers for the long-term monitoring of mechanical parts in high temperature environments. Wei et al. [5] designed an ultrasonic system for ultra-high temperature measurements using materials with a very high melting point as the delay line. Waveguides can also be used to make simultaneous measurements of several material properties. With different waveguide designs and the use of different wave modes, they are easy to be customised and used for a wide range of applications. For example, several researchers use torsional waves to measure the viscosity and density of fluids with waveguides [6,7,8,9,10,11]. Lynnworth [6] first proposed a method to measure material properties, such as liquid density and liquid level, using torsional waves. Kim and Bau [8] further developed this approach to measure both the density and viscosity of a liquid using two waveguides of rectangular and circular geometries, respectively. Similar work were carried out by Shepard et al. [11] and Rabani et al. [10], where magnetostrictive and piezoelectric transducers were used for the excitation and reception of the torsional wave, respectively. More recently Xie, Huan and Li [12] proposed a rapid and reliable torsional resonance-based method for shear modulus measurements using a piezoelectric torsional transducer bonded on a cylindrical specimen. Some researchers use other wave modes in waveguides for fluid property measurements [13,14,15]. Kazys et al. [13] designed a viscosity sensor that consist of an aluminium waveguide in a pipe where shear horizontal (SH) waves are excited from one end and received on the other end. The design was proven suitable for inline monitoring of highly viscous fluids. Cegla et al. [14] reported a method using the ‘quasi-Scholte’ mode on a waveguide dipped in a fluid to measure its bulk velocity and attenuation. Periyannan et al. [16] used the longitudinal mode in waveguides with multiple bent features for simultaneous temperature measurements at different locations.

Most of the applications discussed above can be described as ‘dipstick’ methods where the waveguide is partly or completely immersed in the fluid and the fluid properties are determined by measurements of the ultrasonic properties of the waveguide. Recently, it has been reported that a temperature-sensing accuracy as good as ±0.015 °C can be achieved using SH waves in a ‘dipstick’ waveguide [15]. This is better than the accuracy of most conventional industrial RTDs. The focus of this paper is to further investigate this ‘dipstick’ method and apply it to viscous fluids. A feasibility study is carried out for the simultaneous measurements of fluid temperature and viscosity using a waveguide that operates in the megahertz frequency range. An iterative method is proposed to correct the temperature prediction due to the viscous loading effect on the waveguide which slows down the velocity of the excited travelling wave. This refined ‘dipstick’ method is compared to a highly precise in-lab viscometer (SVM 3001, Anton Parr, Graz, Austria) and a commercial 1/10 DIN PT100 RTD (SE012, Pico Technology, St Neots, UK) in terms of viscosity and temperature measurements, respectively.

## 2. Materials and Methods

Reference measurements with the waveguide were initially performed to obtain ultrasonic wave attenuation and SH wave velocity in the waveguide as a function of temperature using a low-viscosity standard that has a marginal viscosity change as a function of temperature (<1.6 cSt over a temperature change of 50 °C). The viscosity standard chosen for this was the S3S high temperature viscosity standard by Paragon Scientific Ltd. (Prenton, UK). The N35 and N350 general viscosity standards, also purchased from Paragon Scientific Ltd., were used to evaluate the viscosity and temperature measurements using the same waveguide and the results were compared to the Anton Parr viscometer SVM 3001 and the PT100 RTD, respectively.

**Figure 1:**
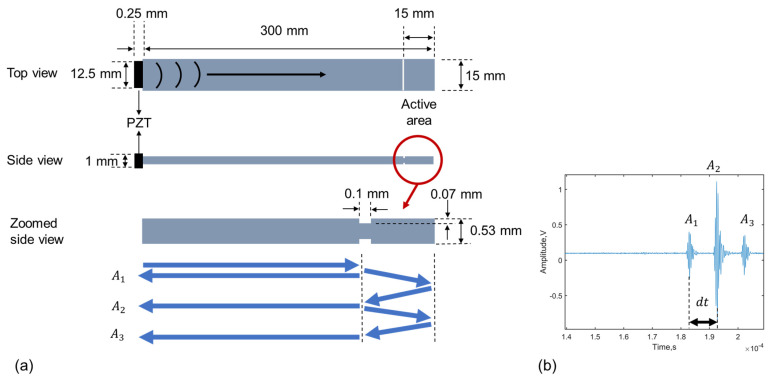
(**a**) Schematic diagram of the waveguide used in this study demonstrating the propagation of ultrasonic wave including the first, second and third echoes; and (**b**) an example of a received signal, indicating the arrival time difference between the echoes.

The waveguide used in this study was made of aluminium with the geometry as shown in Figure 1a. It is a long and thin strip with a piezo-electric transducer (PZT) bonded to one end and identical engraved notches on the front and back surfaces at a distance of 15 mm from the other end. The transducer produces an elastic SH wave that propagates along the waveguide. Lower-order SH waves have been extensively used for structural health monitoring and non-destructive evaluation due to their non-dispersive nature [17]. The nature and characteristics of this mode have been previously investigated and it has been reported that at 2 MHz this wave mode travels non-dispersively (without excessive signal distortion) and with most of its energy concentrated in the centre of the waveguide [18]. This makes it easy to mechanically manipulate the strip via holding onto its edges without affecting the wave propagation.

The wave propagation in the waveguide is as follows: the transducer excites the wave that travels along the waveguide. Once it reaches the notches near the other end, the wave is partially reflected, and this part of the wave travels back and is received by the transducer as the first echo ($A_{1}$). The rest of the wave continues to travel along the waveguide until it reaches the end where it is completely reflected. On the return journey, the wave is again partially reflected by the notches. The remaining part of the wave is transmitted and travels to the transducer to be received as the second echo ($A_{2}$). The reflected part continues to reverberate between the notches and the waveguide end until no more energy remains. These reverberations are received by the transducer as the third echo ($A_{3}$), fourth echo and so on. The ‘active area’ or ‘measurement section’ between the notches and the waveguide end needs to be fully immersed in the fluid sample for a correct measurement. Due the small thickness and the good thermal conductivity of the waveguide material, the active area has a fast response to temperature changes in the surrounding fluid. Thus, the wave velocity in this active area can be used to estimate the temperature of the surrounding fluid sample. As shown in Figure 1b, the time of flight between the first and second reflected signal dt can be used to calculate the shear wave velocity c, knowing that the propagation distance is twice the length of the active area x. This relationship can be expressed by the following equation:

(1) $$c=\frac{2x}{dt}.$$

The shear properties of a fluid can be determined by measuring the attenuation of the SH wave in the waveguide that interacts with it. The attenuation that is purely caused by the leakage of the shear wave into the surround fluid can be estimated by the following equation [19]:

(2) $$\alpha_{s}=\frac{1}{2h}\sqrt{\frac{2{\omega \rho }_{f}\eta }{\rho_{s}G}},$$

where $\alpha_{s}$ is the attenuation of the wave in the waveguide due to shear leakage, h is the thickness of the waveguide, ω is the angular frequency of the transmitted wave, $\rho_{s}$ is the density of the waveguide, G is the shear modulus of the waveguide, $\rho_{f}$ is the density of the fluid and η is the dynamic viscosity of the fluid. The equation above is an approximate formula for SH-wave attenuation and its full derivation and validation are shown in previous work [19,20].

In the past, this ‘shear attenuation’ was measured by immersing a waveguide at two different depths into the fluid sample [14]. However, this method reportedly introduces errors to the measurements, and it is also not ideal for practical online monitoring. By creating notches on the waveguide, it is possible to estimate the attenuation due to shear leakage regardless of the immersion depth. This is achieved by comparing a measurement to a calibration measurement in a reference fluid.

**Figure 2:**
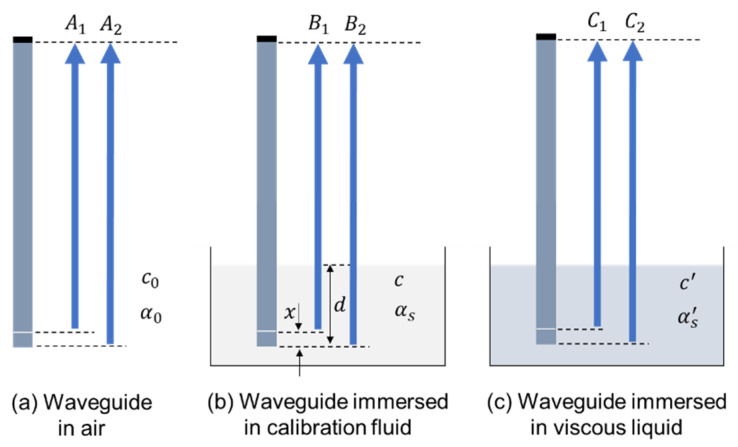
Schematic diagram showing the signal difference in a waveguide (**a**) in air; (**b**) immersed in calibration fluid and (**c**) immersed in a viscous liquid.

The amplitude ratio of the first and second reflections is used to determine the attenuation of the SH wave. As shown in Figure 2a, without any fluid loading, this quantity can be expressed in the form of ‘attenuation’ as

(3) $$\alpha_{0}=-\frac{\ln\left(\frac{A_{1}}{A_{2}}\right)}{2x}.$$

This quantity is a result of the reflection coefficient from the notch and the end of the waveguide and the material attenuation in the waveguide and should be constant as a function of temperature. However, it is impractical to perform reference measurements in air, especially at different temperatures for velocity calibration; therefore, in our experiments the waveguide is immersed in a low-viscosity calibration liquid. When the waveguide is immersed to a depth of d in the calibration fluid, as shown in Figure 2b, additional attenuation induced by the viscous fluid loading should also be considered; thus, this quantity becomes:

(4) $$\alpha \left(T\right)=-\frac{\ln\left(\frac{B_{1}}{B_{2}}\right)}{2x}=\frac{-\ln\left(\frac{A_{1}e^{-\alpha_{s}\times 2\left(d-x\right)}}{A_{2}e^{-\alpha_{s}\times 2d}}\right)}{2x}=\alpha_{0}-\alpha_{s}\left(T\right),$$

where *T* indicates the temperature dependence. Similarly, in a fluid with unknown viscosity as shown in Figure 2c,

(5) $$\alpha^{\prime }\left(T\right)=-\frac{\ln\left(\frac{C_{1}}{C_{2}}\right)}{2x}=\alpha_{0}-\alpha_{s}^{\prime }\left(T\right).$$

The shear leakage-induced attenuation in this case can be determined by comparing Equations (4) and (5):

(6) $$\alpha_{s}^{\prime }\left(T\right)=\alpha \left(T\right)+\alpha_{s}\left(T\right)-\alpha^{\prime }\left(T\right).$$

Even though a low-viscosity standard was chosen for calibration, α is temperature dependent due to $\alpha_{s}$. As the viscosity and density at different temperatures are known for the calibration fluid, $\alpha_{s}$ can be easily calculated using Equation (2). In the measurements for the two viscous liquid samples that were used in this study, we assume that the density does not change as a function of temperature. While this is not always a reasonable assumption, it can be made in this case as the density variations as a function of temperature are small, compared to the change in viscosity over the same temperature range, and the different viscosity standards have very similar density/temperature characteristics.

**Figure 3:**
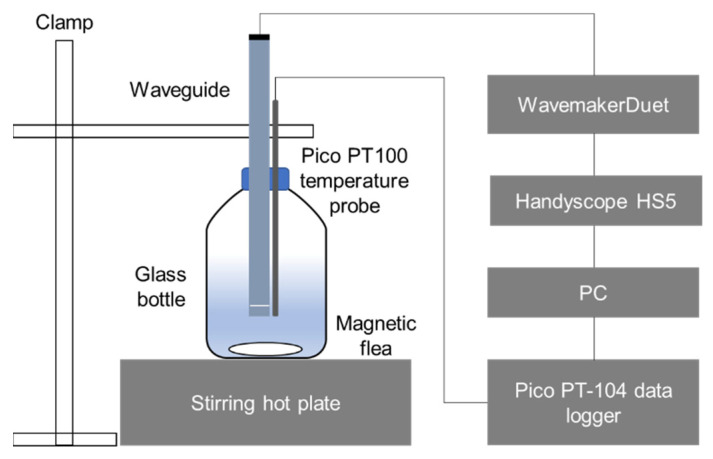
Schematic diagram of the experimental setup.

The experimental setup for the calibration and measurements is shown in Figure 3. The temperature was measured using a PT100 temperature probe and recorded by a platinum resistance data logger (PT-104, Pico Technology, St Neots, UK). An arbitrary function generator/oscilloscope (HandyscopeHS5, TiePie Engineering, Sneek, The Netherlands) was used for the excitation and receiving of the signal with an amplifier (WavemakerDuet by Macro Design Ltd., London, UK). The received signal was amplified by 40 dB and sampled at a frequency of 50 MHz.

Initially, the sample was heated to 90 °C in a glass bottle on top of a hot plate (PC-620D, Corning Inc., New York, NY, USA) with an active magnetic flea. The heater was then switched off and the sample was left to cool down at room temperature. Data collection of the ultrasonic signal and temperature was carried out in one-minute intervals. For each measurement, 400 consecutive signals were sent, received and averaged to improve the signal to noise ratio (SNR). For a typical measurement, a 5-cycle Hann-windowed sinusoidal tone-burst with a centre frequency of 2 MHz was sent to the waveguide and the following data processing methods were used to extract the time of flight and amplitude of the first and second reflections:

- The averaged signal is filtered by a 5th-order Butterworth bandpass filter with cut off frequency at 1.6 and 2.4 MHz;
- The filtered signal is up-sampled to 1 GHz;
- The peaks of the first and second reflections are used to determine the time of flight and the exact locations of the peaks are extracted by gradient-based linear interpolation. The amplitude of the peaks is used to evaluate SH wave attenuation.

Initially, the waveguide was calibrated in Paragon S3S at an elevated temperature with the PT100 probe to obtain two calibration equations, T=f(c) and α=g(T), which represent the linear relationship between the measured wave velocity and temperature and that between the relative amplitude change (i.e., the amplitude ratio of first and second reflections) and temperature, respectively. When the waveguide is used to measure viscous fluids, such as the Paragon N35 or Paragon N350, the measured wave velocity is reduced due to viscous loading of the fluid. Such ‘viscous effects’ of fluids on the propagation of the guided waves have been reported [6,7,21,22], where theories were proposed to describe such behaviour. In such a viscous environment, the viscous boundary layer of the fluid that surrounds the waveguide contributes additional inertia to the waveguide [23]. Thus, to correct the propagation velocity, the viscous effect can be treated as the added effective mass of the viscous boundary layer that is attached onto the waveguide.

The velocity of the shear waves in the unloaded waveguide $c_{0}$ is related to its shear modulus G and density $\rho_{s}$ by the following equation:

(7) $$c_{0}=\sqrt{\frac{G}{\rho_{s}}}.$$

**Figure 4:**
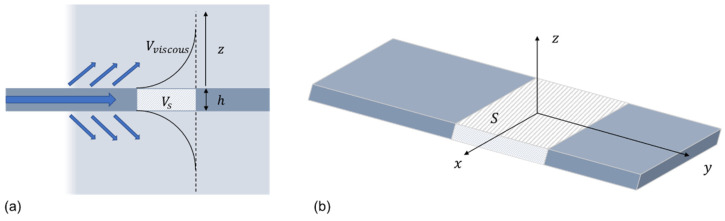
Schematic diagram showing the viscous loading of the surrounding fluid on the waveguide as the SH wave propagates and part of it leaks into the fluid: (**a**) 2D view from the y–z plane; and (**b**) 3D view.

As Figure 4a shows, the shear wave motion into the adjacent fluid is exponentially decreasing with distance away from the waveguide surface. For a Newtonian fluid, this characteristic layer has a thickness of

(8) $$\delta =\sqrt{\frac{2\nu }{\omega }},$$

where ν is the kinematic viscosity of the fluid and ω is the angular frequency of the shear wave.

Consider a section of the immersed waveguide as shown in Figure 4b, where the area of the waveguide surface on the x–y plane is S, the thickness of the waveguide is h and the wave propagates towards y; the volume that is occupied by the fluid in the viscous skin depth, as shown in Figure 4a, can be calculated by

(9) $$V_{\mathrm{viscous}}=S\delta .$$

For an oscillating plate loaded with a viscous fluid, only half of the mass of the viscous skin depth contributes to the inertia of the plate [24]. The effective density $\rho_{eff}$ of the fluid-loaded waveguide can therefore be expressed by adding this additional mass contribution on both sides of the waveguide with a thickness of half the viscous skin depth:

(10) $$\rho_{eff}=\frac{\rho_{s}V_{s}+2\rho_{f}(\frac{1}{2}V_{\mathrm{viscous}})}{V_{s}},$$

where $\rho_{s}$ and $\rho_{f}$ are the densities of the waveguide and the fluid. Here, $V_{s}$ is the volume of the section of waveguide being referred to and this can be calculated by

(11) $$V_{s}=Sh.$$

The shear velocity in the viscous fluid loaded waveguide with effective density will be

(12) $$c_{loaded}=\sqrt{\frac{G}{\rho_{eff}}.}$$

By substituting Equations (8)–(11) into (12) and rearranging, the wave velocity for a waveguide with viscous layer loading can be calculated as

(13) $$c_{loaded}=\sqrt{\frac{c_{0}^{2}}{1+\frac{{\delta \rho }_{f}}{h\rho_{s}}}.}$$

**Figure 5:**
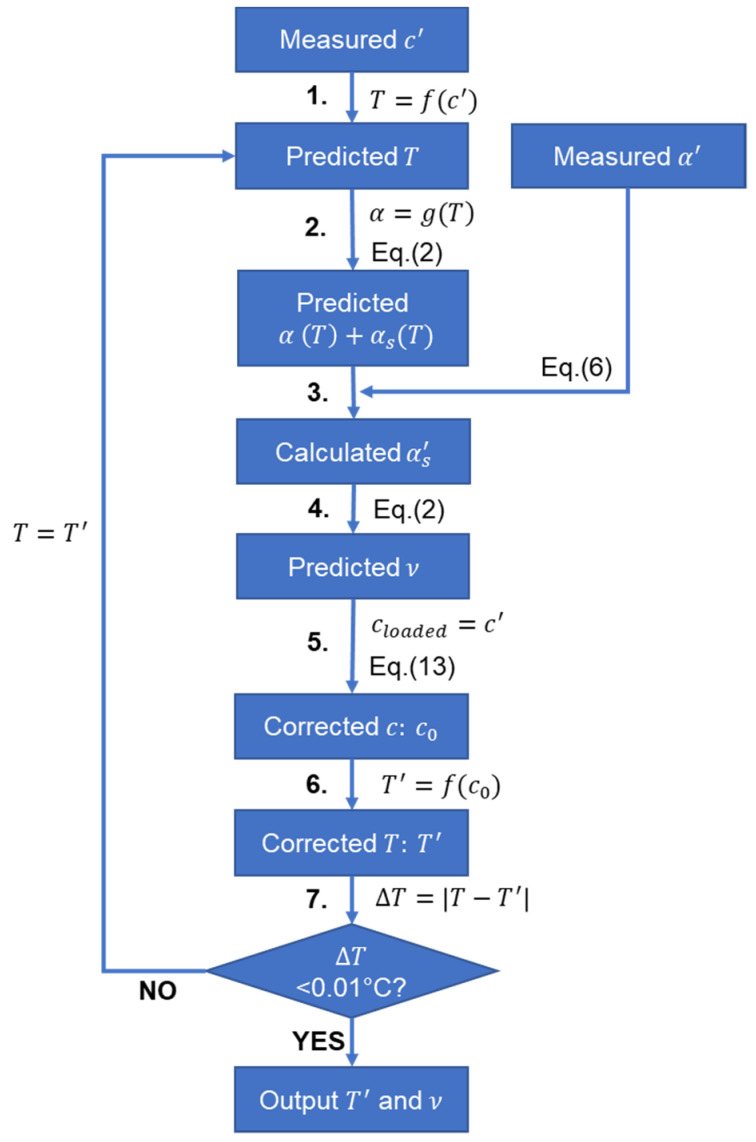
Iteration algorithm for temperature prediction correction based on the measured attenuation due to shear leakage with the effect of the viscous loading by the surrounding fluid.

The algorithm shown in Figure 5 is used to correct the temperature predicted for viscous fluids with the calibration data collected using a low-viscosity liquid. Initially (Step 1), the measured shear wave velocity is used to predict the fluid temperature using the temperature velocity calibration equation $T=f\left(c^{\prime }\right)$, assuming no viscous loading on the waveguide. This temperature is then used (in Step 2) to obtain reference α(T) and $\alpha_{S}\left(T\right)$, which are used (in Step 3) to calculate the shear wave attenuation for the viscous sample $\alpha_{S}^{\prime }$ with the measured $\alpha^{\prime }$. Following this, in Step 4, the viscosity of the fluid can be easily estimated using Equation (2), which is then used (in Step 5) to correct the shear wave velocity with Equation (13). The correction here gives an estimate of the ‘true wave velocity’ if there was no viscous loading. The corrected velocity then updates the temperature prediction (in Step 6), which in turn corrects the calibration point for shear wave attenuation (i.e., back to Step 2 in another iteration) and therefore further improves the viscosity prediction. This is repeated until the temperature outputs between the two iterations have a difference smaller than 0.01 °C (Step 7).

## 3. Results and Discussion

**Figure 6:**
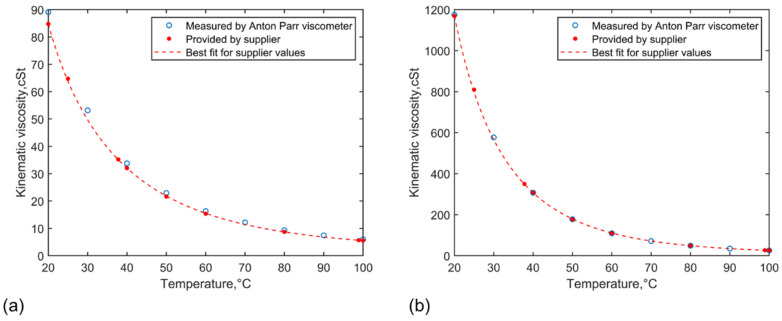
Results of the kinematic viscosity measured by the Anton Parr viscometer plotted against temperature, compared with the values provided by the viscosity standard supplier for (**a**) Paragon N35 and (**b**) Paragon N350.

Viscosity measurements were carried out to validate the viscosity standards—Paragon N35 and N350—in the temperature range of 20 to 100 °C in 10 °C intervals. As Figure 6 shows, the kinematic viscosity results are plotted against temperature in comparison with the values provided by the supplier. The Anton Parr VM3001 is a highly precise in-lab viscometer that has a repeatability of 0.1% in viscosity. The results produced by the Anton Parr VM3001 are in excellent agreement with the values provided by the supplier for both viscosity standards with a small offset at the lower temperature end for Paragon N35. The root mean square (rms) of the difference between the measured value by the Anton Parr viscometer at each temperature and the value given by the best line fit for the supplier values at the same temperature is 1.54 cSt for Paragon N35 and it is 2.28 cSt for Paragon N350. If the difference is divided by the value given by the best line fit, the calculated mean percentage error will be 6.47% for Paragon N35 and 0.65% for Paragon N350.

**Figure 7:**
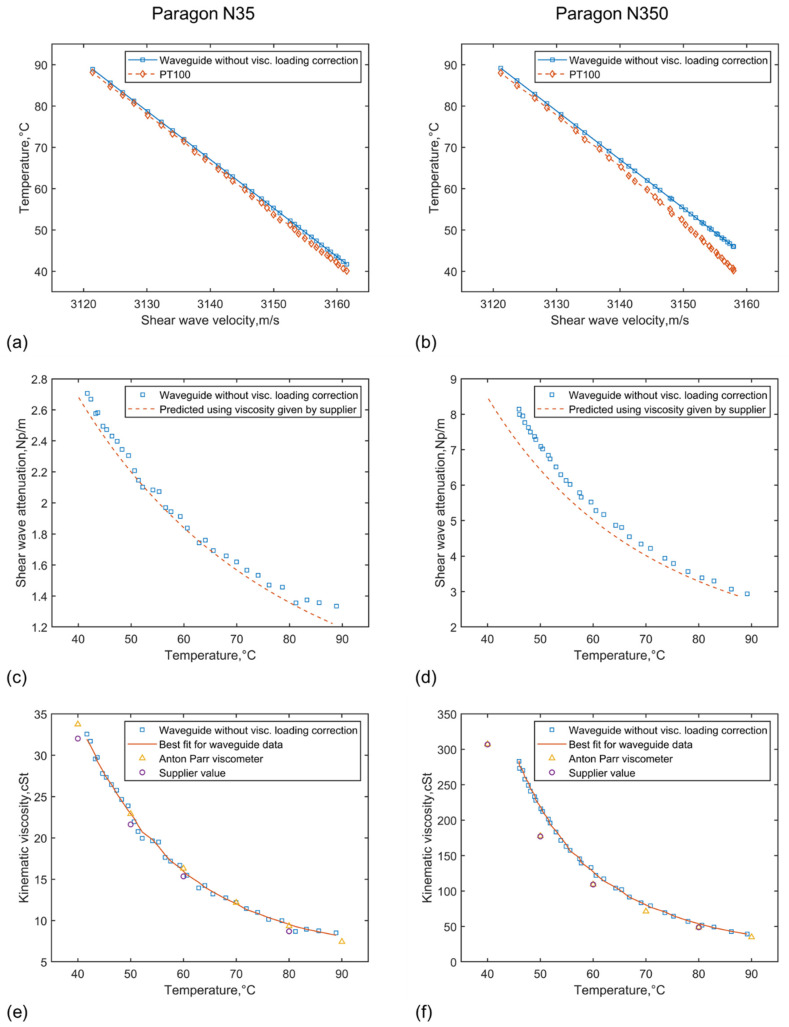
Temperature predicted, attenuation measured and viscosity predicted for Paragon N35 (**a**,**c**,**e**) and Paragon N350 (**b**,**d**,**f**), where the temperature is compared with measurements with PT100, the attenuation is compared with that predicted using the kinematic viscosity provided by the viscosity standard supplier, and the viscosity predicted is compared with measurements using the Anton Parr viscometer and the values provided by the viscosity standard supplier (results for predictions without correcting for viscous fluid loading effect on the wave velocity).

The waveguide measurements were also carried out for these two viscosity standards. The results in Figure 7 show outcomes when no temperature/velocity correction is applied. Here, the data were collected for the waveguide and down-sampled from three repeated cycles of heating and cooling in 1-minute intervals in the temperature range of 40 to 90 °C. The predicted shear attenuation was calculated using the viscosity values provided by the supplier of the viscosity standards. The density of the fluid was assumed to be constant (850 kg/m^3^) while the density of the waveguide was assumed to be 2690 kg/m^3^ and its shear modulus 26.34 GPa.

As can be seen from Figure 7a, the waveguide seems to overpredict the temperature towards the lower temperature end over the range of 40 to 90 °C. Similar trends in overestimates can be observed for N350, as shown in Figure 7b, but with a larger increase in the over-prediction at a lower temperature. In both cases, the offset in the temperature measurements between the waveguide and the PT100 is related to the viscous loading from the surrounding fluid. The over-prediction in temperature is due to the reduction in shear velocity as a result of viscous fluid loading compared to measurements in the very low, practically non-viscous calibration fluid. As explained earlier, the effect of the viscous loading on the waveguide can be interpreted as the addition of the mass of an effective layer of the fluid to the overall waveguide material mass. This adds inertia to the waveguide and slows down the shear wave propagation.

As a result of this over-prediction of temperature, there is an offset in the attenuation measured and therefore the viscosity predicted in Figure 7c–f. For Paragon N35, the predicted viscosity seems to still agree with that determined by the Anton Parr viscometer as well as the values provided by the viscosity standard supplier with a negligible divergence (follows Anton Parr viscometer), while for N350 the over-prediction in temperature results in an over-estimated viscosity by almost as much as 50 cSt in the lower temperature limit (when the viscosity is high) compared with other methods.

**Figure 8:**
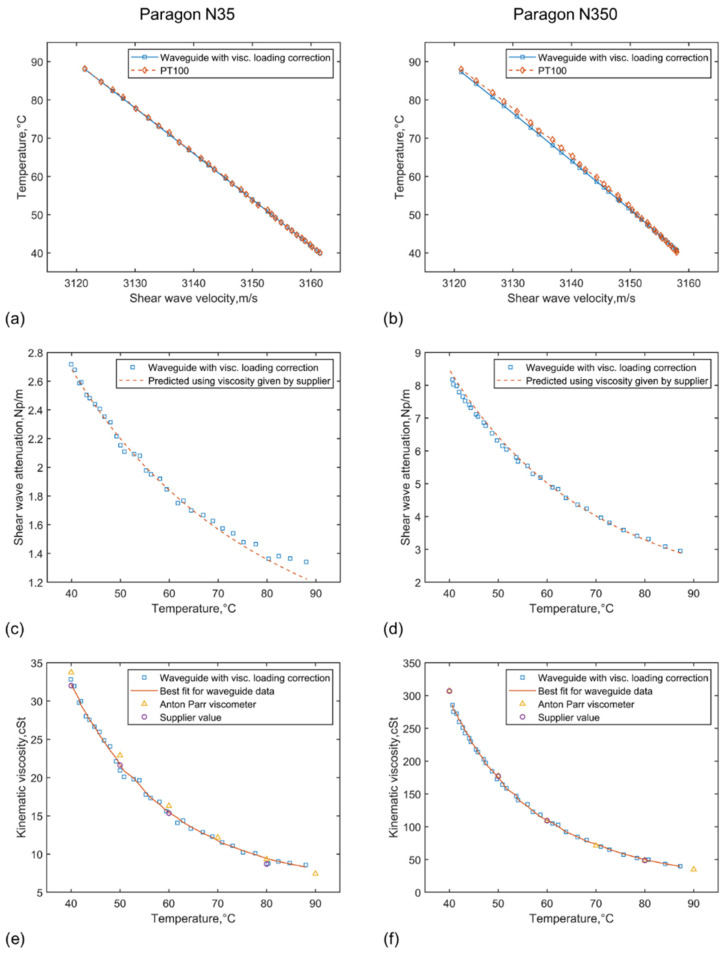
Temperature, attenuation and viscosity predictions corrected using the literation algorithm for Paragon N35 (**a**,**c**,**e**) and Paragon N350 (**b**,**d**,**f**), where the temperature is compared with measurements with PT100, the attenuation is compared with that predicted using the kinematic viscosity provided by the viscosity standard supplier, and the viscosity predicted is compared with measurements using the Anton Parr viscometer and the values provided by the viscosity standard supplier (Results for predictions with correcting for viscous fluid loading effect on the wave velocity).

In Figure 8, the temperature prediction has been corrected following the iteration algorithm described earlier and this in turn corrects/improves the viscosity predicted. As can be seen in Figure 8a,b, the temperature predicted is now in excellent agreement with the PT100. As a result of that, the viscosity prediction is further improved for the measurements for Paragon N35. The predicted values by the waveguide appear to have a closer match to the those provided by the supplier in this case. For Paragon N350, the corrected viscosity prediction is also in excellent agreement with both the Anton Parr measurements and the viscosity standard supplier values.

The repeatability is calculated here as the root mean square of the variance between the measured values and the least square best line fit over the whole range of viscosity/temperature studied. For Paragon N35, it is estimated to 1.80% on average and 0.05 °C (or 0.1%) for temperature prediction. For Paragon N350, a repeatability of 1.17% is achieved for viscosity prediction and 0.20 °C (or 0.4%) for temperature prediction.

There are a number of sources of errors in this study and the following areas are considered the main contributions: (1) uncertainties in the physical properties of the waveguide and the fluids; (2) the instrumental errors in the temperature measurements by the PT100 and the ultrasonic measurements using the pulser/amplifier (non-linearities); and (3) wave modes other than SH excited by the transducer. While the data presented here demonstrate very good agreement (e.g., agreement between waveguide data and that produced by Anton Parr viscometer), the viscosity is predicted using the physical properties (i.e., density and shear modulus), which are calculated with reasonable assumptions or taken directly from the literature rather than experimentally characterised. While the density and viscosity are explicitly known for the calibration fluid and used to calculate $\alpha_{s}$, in the measurements of the test fluids (Paragon N35 and N350), the fluid density is assumed in order to calculate the viscosity from the measured attenuation of the SH wave. Although as stated earlier, the variation in density is small compared to viscosity in response to temperature change, this assumption does introduce some error. The thickness of the waveguide is also nominal only and a tolerance of up to 0.05 mm can be estimated. The pathlength between two reflected signals is calculated as twice the separation between the notch and the tip which is expected to be 15 mm but subject to human error during manufacturing. The commercial 1/10 DIN RTD PT100 temperature sensor was used for calibrating the waveguide but it has an instrumental error varying from ±0.06 to ±0.11 °C in the temperature range of 40 to 90 °C. The ultrasonic signal excited by the transducer is expected to be an SH wave propagating throughout the rectangular geometry. However, the transducer is not perfect and other guided wave modes will be excited at lower amplitudes. These can affect the measured signals and therefore introduce errors into the temperature and viscosity predictions. This can be minimised by careful design and manufacturing of the transducer and the waveguide.

## 4. Conclusions

This paper presents a method to use an ultrasonic waveguide to simultaneously measure the temperature and viscosity of viscous fluids. Unprecedented measurement precision for this type of ‘dipstick’ setup is reported and the velocity estimates are so precise that for the first time the effect of viscous fluid loading onto the waveguide is demonstrated. An iterative algorithm to correct for the changes in wave velocity due to viscous mass loading was presented and shown to considerably correct the temperature measurements and improve the viscosity predictions. The overall results predicted are in excellent agreement with reference measurements using an Anton Parr viscometer, a PT104 RTD for temperature measurements and literature values provided by the viscosity standard supplier. The observed repeatability was less than 2.0% in kinematic viscosity in the range from around 8 cSt up to 300 cSt and less than 0.5% in temperature from 40 to 90 °C. Without the correction for velocity, the temperature prediction could have an error as large as 15% which would divert the viscosity prediction as much as about 15% for a fluid that has a similar viscosity as the Paragon N350. The refined ‘dipstick’ method reported here gives reliable and accurate measurements for both temperature and viscosity.

## Data Availability

The data presented in this study are available on request from the corresponding author.
